# Supplementary material for: A Critical View of the Application of the APEX Software (Aqueous Photochemistry of Environmentally-Occurring Xenobiotics) to Predict Photoreaction Kinetics in Surface Freshwaters
Source: Molecules. 2019 Dec 18;25(1):9. doi: 10.3390/molecules25010009 (PMC7017383; doi:10.3390/molecules25010009)
Supplement: Supplementary file 1 [file molecules-25-00009-s001.zip › 3.2.4_gcc-4.4.0.zip!/3.2.4_gcc-4.4.0/3.2.4_gcc-4.4.0/doc/octave/html/interpreter/Expressions-Involving-Permutation-Matrices.html]

Expressions Involving Permutation Matrices - Untitled


Previous: Expressions Involving Diagonal Matrices,
Up: Matrix Algebra


---

#### 20.2.2 Expressions Involving Permutation Matrices

If P is a permutation matrix and M a matrix, the expression
`P*M` will permute the rows of M. Similarly, `M*P` will
yield a column permutation.
Matrix division `P\M` and `M/P` can be used to do inverse permutation.

The previously described syntax for creating permutation matrices can actually
help an user to understand the connection between a permutation matrix and
a permuting vector. Namely, the following holds, where `I = eye (n)`
is an identity matrix:

```
       I(p,:) * M = (I*M) (p,:) = M(p,:)
```

Similarly,

```
       M * I(:,p) = (M*I) (:,p) = M(:,p)
```

The expressions `I(p,:)` and `I(:,p)` are permutation matrices.

A permutation matrix can be transposed (or conjugate-transposed, which is the
same, because a permutation matrix is never complex), inverting the
permutation, or equivalently, turning a row-permutation matrix into a
column-permutation one. For permutation matrices, transpose is equivalent to
inversion, thus `P\M` is equivalent to `P'*M`. Transpose of a
permutation matrix (or inverse) is a constant-time operation, flipping only a
flag internally, and thus the choice between the two above equivalent
expressions for inverse permuting is completely up to the user's taste.

Multiplication and division by permutation matrices works efficiently also when
combined with sparse matrices, i.e., `P*S`, where P is a permutation
matrix and S is a sparse matrix permutes the rows of the sparse matrix and
returns a sparse matrix. The expressions `S*P`, `P\S`, `S/P` work
analogically.

Two permutation matrices can be multiplied or divided (if their sizes match), performing
a composition of permutations. Also a permutation matrix can be indexed by a permutation
vector (or two vectors), giving again a permutation matrix.
Any other operations do not generally yield a permutation matrix and will thus
trigger the implicit conversion.
